# Identifying dietary and daily product-use patterns explaining joint variation in multiple urinary phthalate metabolites via sparse reduced-rank regression

**DOI:** 10.3389/fnut.2026.1929257

**Published:** 2026-09-22

**Authors:** Hyun-Wook Park, Donghwan Lee, Bomi Park

**Affiliations:** 1 Department of Preventive Medicine, College of Medicine, Chung-Ang University, Seoul, Republic of Korea; 2 Department of Statistics, Ewha Womans University, Seoul, Republic of Korea

**Keywords:** biomonitoring, dietary exposure, drinking water, exposure assessment, food contact materials, phthalates, sparse reduced-rank regression

## Abstract

**Background:**

Phthalates are synthetic plasticizers widely used in consumer and industrial products, resulting in widespread human exposure and potential adverse reproductive, neurodevelopmental, and metabolic effects. However, the relative contributions of diverse lifestyle-related sources to the overall phthalate burden remain unclear. In this study, we aimed to identify dietary and daily product use factors most prominently associated with joint variations in urinary phthalate metabolite mixtures in a nationally representative sample of Korean adults.

**Methods:**

We analyzed data from 3,760 adults from the Korean National Environmental Health Survey Cycle 4 (2018–2020). Information on dietary consumption, food-contact material use, and consumer product use was collected through questionnaires, and urinary concentrations of eight phthalate metabolites [mono-methyl phthalate, mono-ethyl phthalate, mono-*n*-butyl phthalate, mono-benzyl phthalate, mono-(2-ethyl-5-hydroxyhexyl) phthalate, mono-(2-ethyl-5-oxohexyl) phthalate, mono-(2-ethyl-5-carboxypentyl) phthalate, and mono-(3-carboxypropyl) phthalate] were measured. Sparse reduced-rank regression was used to identify latent patterns of dietary consumption, food-contact material use, and consumer product use jointly associated with the eight metabolite levels after covariates adjustment, and analyses were repeated by sex.

**Results:**

A single latent pattern was identified, in which all phthalate metabolites showed positive loadings (+0.10 to +0.46). Mineral spring water (+0.08), grains (+0.08), tap water (+0.06), and beverages in plastic pouches (+0.08) exhibited the largest loadings in the same direction as the phthalate metabolites. In sex-stratified analyses, drinking water and grain consumption among men and the use of beverages in plastic pouches and makeup products among women showed prominent loadings in the same direction as the eight metabolites.

**Conclusion:**

Consumption of mineral spring water, tap water, grains, and beverages in plastic pouches were major dietary and daily product use factors associated with higher urinary phthalate metabolite mixtures in Korean adults. Given that current Korean regulations cover a limited subset of phthalates at levels less stringent than international standards, expanding regulatory frameworks for drinking water and food-contact materials and adopting precautionary lifestyle measures are warranted, along with prospective studies to clarify source-specific causal pathways.

## 1 Introduction

Phthalates are a group of synthetic chemicals derived from ortho-phthalic acid and are widely used as plasticizers to enhance the flexibility and softness of plastics (1). They are used in a broad range of products, including food-contact materials (FCMs), personal care products, building materials, home improvement and cleaning products, and medical devices (1, 2). Phthalates do not form covalent bonds with plastic polymers; hence, they easily leach into the surrounding environment during production, usage, and disposal stages (3). Consequently, phthalates have become ubiquitous in environmental and biological matrices worldwide (1, 2, 4, 5).

Human exposure to phthalates is therefore widespread, continuous, and mediated through multiple pathways across the general population, posing a substantial public health concern (1–4, 6). Phthalates are recognized as endocrine disruptors and have been associated with adverse reproductive (7, 8) and metabolic outcomes (9) and, although the evidence is less consistent, with neurodevelopmental outcomes (10, 11), at the chronic low-level exposures typical of the general population (5, 12). Accordingly, characterizing the background exposure to phthalates is critical.

Background exposure is typically assessed through human biomonitoring, with biomarker selection guided by phthalate metabolism. Parent phthalate diesters are rapidly hydrolyzed by esterases to form their corresponding primary monoester metabolites. Low-molecular-weight phthalates are excreted mainly as these monoesters, whereas high-molecular-weight phthalates undergo additional side-chain oxidation to secondary metabolites (5, 6, 13–15). Thus, urinary metabolites serve as biomarkers of phthalate exposure. They are preferred over the parent diesters because diesters are ubiquitous in laboratory and sampling materials, making their measurement in blood or serum susceptible to external contamination (6, 13).

Effective risk management requires not only the measurement of phthalate exposure levels but also a clear understanding of how different exposure sources contribute to the overall burden of phthalates. Such knowledge enables prioritization of source-specific interventions, risk communication, and public health policies. Despite the growing body of research, substantial gaps remain in quantifying the relative contributions of different sources to human phthalate exposure in the general population (5, 12). These are further compounded by the diversity of exposure pathways, concurrent exposure to multiple phthalates as mixtures, and their pervasive presence in consumer products (6, 16), suggesting the need for joint evaluation of multiple lifestyle-related exposure sources and diverse phthalate metabolites.

To address these gaps, we employed an outcome-wide analysis to examine how multiple lifestyle determinants contribute to phthalate exposures (17). Using a nationally representative sample of Korean adults, we analyzed dietary consumption and daily product use, which are closely linked to individual lifestyles. Given the widespread, multifactorial, and unavoidable nature of phthalate exposure, we aimed to quantify the relative importance of lifestyle factors in explaining the joint variation in urinary phthalate metabolite mixtures. Our findings are expected to contribute to addressing existing knowledge gaps regarding exposure characterization needed to inform exposure-reduction strategies (18).

## 2 Materials and methods

### 2.1 Data and study participants

This study analyzed publicly available data from the Korean National Environmental Health Survey (KoNEHS) Cycle 4, a nationwide biomonitoring program conducted by the National Institute of Environmental Research (NIER) under the Ministry of Environment from 2018 to 2020. Since 2009, KoNEHS has assessed internal exposure to hazardous environmental chemicals in the general Korean population, with each cycle spanning 3 years.

The sampling design of KoNEHS Cycle 4 has been described in detail elsewhere (19). The target population of the adult survey comprised Korean residents aged 19 years and older, and the census tract list of the 2015 Population and Housing Census served as the sampling frame for the survey. A two-stage stratified design was applied to ensure the representativeness of the national population. Primary stratification was based on administrative regions (provinces, metropolitan cities, and municipalities) and coastal areas delineated by topography, with sites hosting air pollution monitoring stations incorporated to evaluate the relationships between ambient air pollution and health outcomes. Secondary stratification further divided these areas by socioeconomic characteristics, including housing type and residential area features, yielding a total of 69 strata. Survey sites were distributed across strata via square-root proportional allocation to the number of households per stratum–an approach selected because it increases the sampling fraction in less populous provinces, thereby improving the precision of the region-specific estimates. On this basis, approximately 15 adults were targeted at each of the 245 survey sites.

Data collection included structured questionnaires on sociodemographic and lifestyle factors, physical examinations, and blood and urine sampling. Following standardized field protocols, trained interviewers and staff conducted face-to-face interviews, physical measurements, and biospecimen collection. The study protocol was approved by the Institutional Review Board of the NIER (NIER-2018-BR-003-02), and all participants provided written informed consent.

Of the 4,239 adults aged ≥19 years, 443 were excluded because urinary creatinine concentrations were outside the acceptable range of 0.3–3.0 g/L, a criterion adopted by the World Health Organization based on the principle that highly dilute or highly concentrated urine specimens are generally unsuitable for biomonitoring (19, 20), and 36 were excluded owing to missing or incomplete covariate data. The final analytical sample comprised 3,760 participants.

### 2.2 Urinary phthalate metabolite levels

Phthalate exposure was assessed by measuring urinary concentrations of the following metabolites: mono-methyl phthalate (MMP), mono-ethyl phthalate (MEP), mono-*n*-butyl phthalate (MnBP), mono-benzyl phthalate (MBzP), mono-(2-ethyl-5-hydroxyhexyl) phthalate (MEHHP), mono-(2-ethyl-5-oxohexyl) phthalate (MEOHP), mono-(2-ethyl-5-carboxypentyl) phthalate (MECPP), and mono-(3-carboxypropyl) phthalate (MCPP). Analyses were conducted using an Agilent 1200 series/Agilent 6490 high-performance liquid chromatography–tandem mass spectrometer (HPLC-MS/MS; Agilent, Santa Clara, CA, USA). Sample preparation involved enzymatic deconjugation with β-glucuronidase from *Escherichia coli* K 12.

Detailed procedures for biological specimen management and quality assurance and control protocols have been described elsewhere (19). Quantification was performed using calibration curves prepared by spiking artificial urine with native analytical standards of the eight target metabolites, with ^13^C-labeled analogs as internal standards. Calibration standards were prepared at a minimum of four to five concentration levels spanning the range expected in the general population, and calibration curves were expressed as relative response as a function of relative concentration. The calibration ranges were 1.0–120 μg/L for MEHHP and MEOHP, 2.0–240 μg/L for MEP, MnBP, and MECPP, and 0.5–60 μg/L for MMP, MBzP, and MCPP. Calibration standards were prepared and measured for each analytical batch, and calibration curves were accepted only when R^2^ ≥ 0.995. Calibration verification standards were analyzed after every 20 participant samples and were required to fall within ±15% of their reference values. When this criterion was not met, the calibration curve was reconstructed and the preceding 20 samples were reanalyzed. The limits of detection (LODs) were determined by fortifying pooled or artificial urine at approximately 3–10 times the estimated detection limit and processing seven samples. The LOD was calculated as 3.14 × SD, where SD is the standard deviation of the seven replicate measurements and 3.14 is the t-statistic for six degrees of freedom at a two-sided 98% (equivalently, one-sided 99%) confidence level. The LODs were 0.069 μg/L for MMP, 0.108 μg/L for MEP, 0.084 μg/L for MnBP, 0.030 μg/L for MBzP, 0.069 μg/L for MEHHP, 0.052 μg/L for MEOHP, 0.073 μg/L for MECPP, and 0.072 μg/L for MCPP. Concentrations below the LOD were imputed as LOD/√2 (19). To account for variations in urinary dilution, metabolite concentrations were corrected for creatinine (19). Because these creatinine-corrected concentrations were right-skewed, they were log-transformed prior to statistical analysis.

### 2.3 Dietary and product-use factors in daily life

Information on dietary consumption and the use of FCMs and consumer products was obtained using comprehensive questionnaires (Table 1).

**TABLE 1:** Dietary and daily product-use factors evaluated in the study.

Category	Subcategory	Specific items
Dietary consumption	Drinking water	Mineral spring water
		Tap water
		Filtered water
		Bottled water
	Seafood	Large fish (including tuna)
		Fish
		Crustaceans
		Clams
		Seaweed
		Other seafood
	Plant-based food	Fruits
		Vegetables
		Kimchi
		Grains
		Beans (including bean products)
	Dairy products and meat	Dairy products
		Grilled meat
	Processed food	Cup noodles
		Fast food
		Microwaveable packaged food
		Popcorn
		Canned food
Food contact materials use	Food packaging and containers	Delivery food wrapped in plastic film
		Plastic containers for storing hot food
		Beverages in plastic pouches
		Beverages in polyethylene terephthalate bottles
		Disposable paper cups
	Non-stick cookware	Coated containers
		Coated frying pans
		Coated pots
		Coated rice cookers
Consumer products use	Personal care products	Toothbrushes
		Perfumes
		Hair products
		Body wash
		Makeup products
		Nail polish
		Antimicrobial products
	Cleaning products	Air fresheners
		Fabric cleaners
		Household cleaners

Several variables were excluded for methodological reasons. The use of plastic containers for storing hot boiled water and the use of coatings and polishes were excluded due to low prevalence (approximately 5%), which limited their relevance to routine daily use. Ultraviolet-protective sunscreen use was excluded because KoNEHS, conducted throughout the year, records product use over the preceding 3 months, a measure susceptible to misclassification given the strong seasonality of sunscreen use. After these exclusions, 41 dietary and product-use factors were retained for analysis, comprising 22 dietary items (four drinking-water types, six seafood categories, five plant-based food groups, two dairy and meat items, and five processed-food items), nine food-contact material items (five packaging or container types and four non-stick cookware items), and 10 consumer-product items (seven personal care and three cleaning products).

For drinking water, participants were classified as consumers or non-consumers of each water type based on reported use at home or elsewhere. This approach was adopted because the KoNEHS questionnaire records the main type of water consumed separately for home and out-of-home settings but does not distinguish daily-intake volume by location or type. Therefore, the amount of each specific water type consumed could not be quantified. For other dietary and product-use factors, frequency responses were converted to daily equivalents; for instance, a reported frequency of “4–5 times weekly” was converted to 0.64 times per day (= 4.5/7).

### 2.4 Covariates

Sociodemographic and behavioral factors used as covariates included age, sex, monthly household income, educational attainment, marital status, residential area, smoking status, drinking status, body mass index (BMI), daily indoor time, and recent renovation or repair. Age (years), BMI (kg/m^2^), and daily indoor time (h) were treated as continuous variables, whereas the remaining factors were categorical.

Sex was classified as male or female. Monthly household income was reported in Korean won (KRW) per month across six ranges: <1 million, 1–<2 million, 2–<3 million, 3–<5 million, 5–<7 million, and ≥7 million. Educational attainment was categorized as below high school, high school, or beyond high school. Marital status was classified as married/living with a partner or never married/divorced/separated/widowed. Residential areas were classified as coastal or non-coastal and included as a covariate to account for broad geographic variations, including environmental background, food origins, water infrastructure, and other regional features not fully represented by the individual-level questionnaire variables alone.

Smoking status was defined as current or non-current, with the latter category including both former- and never-smokers. Drinking status was determined based on binge drinking, defined as consuming ≥7 drinks for men and ≥5 drinks for women at least once a month. Recent renovation or repair was recorded if participants reported undertaking such activities in their primary residence within the past 6 months.

### 2.5 Statistical analysis

Statistical analyses were conducted using R software (version 4.5.1). Continuous variables were reported as medians with interquartile ranges (IQRs), and sex differences were evaluated using the Mann–Whitney U test. Categorical variables were summarized as frequencies and percentages, with differences by sex evaluated using chi-square tests.

Urinary creatinine-corrected phthalate metabolite concentrations were described using geometric means (GMs), geometric standard deviations (GSDs), and the 25th, 50th, and 75th percentiles. Sex differences in log-transformed values were examined using two-sample *t*-tests. Pearson’s correlation coefficients were calculated to evaluate correlations among log-transformed values.

To identify latent patterns of dietary consumption and daily product use associated with urinary phthalate metabolite levels, we applied sparse reduced-rank regression (sRRR), a dimension-reduction technique (17). Reduced-rank regression (RRR) derives linear combinations of predictors that explain the maximal variation in the multivariate outcomes (21). By imposing a low-rank structure on the regression coefficient matrix, RRR reduces the number of parameters to be estimated and incorporates outcome correlations to enhance predictive performance. To improve stability under predictor collinearity and facilitate variable selection, we used sRRR, a penalized extension of RRR that shrinks coefficients of less informative predictors toward zero while retaining the predictors that contribute most strongly to the outcome structure (22, 23).

In sRRR, dimensionality reduction is achieved by imposing a low-rank constraint on the coefficient matrix, which is factorized as *C* = UDV*^T^* through singular value decomposition (SVD). Each rank corresponds to a latent pattern, defined as a linear combination of predictors jointly explaining outcome variation. The corresponding singular values, given by the diagonal entries of D, reflect the relative importance of each latent pattern. The left singular vectors (columns of U) and right singular vectors (columns of V) represent predictor and outcome loadings, respectively. The absolute value of each loading indicates the strength of the corresponding variable’s association with the latent pattern, and its sign denotes the direction. Because SVD is subject to sign indeterminacy, simultaneously reversing the signs of corresponding columns of U and V does not alter the reconstructed coefficient matrix or the relationships between variables. Accordingly, signs were oriented so that most outcome loadings were positive, facilitating interpretation. Predictors with loadings sharing the same sign as the outcome loadings indicate positive contributions to the corresponding metabolite concentrations. The sRRR analysis was conducted using the rrpack package (24).

All variables were centered and scaled before modeling. Categorical covariates were converted into ordinal scales by assigning increasing integer values to represent higher category levels. Covariates were adjusted by exempting them from penalties during model fitting (17), ensuring that latent patterns reflected dietary and daily product-use factors after adjustment for confounders. Model selection involved determining the optimal rank based on the Bayesian information criterion. Because sex may influence exposure patterns and metabolite levels, subgroup analyses were stratified by sex.

## 3 Results

### 3.1 Study population

The sociodemographic and behavioral characteristics of the 3,760 participants are presented in Table 2. The median age was 54 years (IQR: 41–64 years), and women comprised 53.0% of the sample. The most common monthly household income was 3–5 million KRW. Educational attainment beyond high school was reported by 43.5% of the participants, whereas 74.9% were never married, divorced, separated, or widowed. Men had a significantly higher proportion of individuals with higher educational attainment than women, and women had a significantly higher proportion of married individuals than men (*p* < 0.01). Most participants (95.0%) resided in non-coastal areas.

**TABLE 2:** Characteristics of the study participants.

Characteristics	Total (*n* = 3,760)	Male (*n* = 1,766)	Female (*n* = 1,994)	*P*-value
Age (years)^a^	54 (41–64)	54 (41–65)	54 (41–63)	0.11
Household income (KRW/month)^b^				0.19
<1	514 (13.7)	228 (12.9)	286 (14.3)	
1–2 million	573 (15.2)	253 (14.3)	320 (16.0)	
2–3 million	643 (17.1)	304 (17.2)	339 (17.0)	
3–5 million	1,008 (26.8)	503 (28.5)	505 (25.3)	
5–7 million	625 (16.6)	286 (16.2)	339 (17.0)	
≥7 million	397 (10.6)	192 (10.9)	205 (10.3)	
Educational attainment^b^				<0.01
<High school	981 (26.1)	367 (20.8)	614 (30.8)	
High school	1,142 (30.4)	561 (31.8)	581 (29.1)	
>High school	1,637 (43.5)	838 (47.5)	799 (40.1)	
Marital status^b^				<0.01
Never married, divorced, separated, or widowed	2,817 (74.9)	1,369 (77.5)	1,448 (72.6)	
Married, or living with a partner	943 (25.1)	397 (22.5)	546 (27.4)	
Residential location^b^				0.98
Non-coastal	3,572 (95.0)	1,677 (95.0)	1,895 (95.0)	
Coastal	188 (5.0)	89 (5.0)	99 (5.0)	
Smoking^b^				<0.01
No	3,122 (83.0)	1,189 (67.3)	1,933 (96.9)	
Yes	638 (17.0)	577 (32.7)	61 (3.1)	
Drinking^b^				<0.01
No	2,798 (74.4)	1,071 (60.6)	1,727 (86.6)	
Yes	962 (25.6)	695 (39.4)	267 (13.4)	
BMI (kg/m^2^)^a^	24.6 (22.4–26.9)	25.1 (23.2–27.3)	23.9 (21.7–26.5)	<0.01
Indoor time (h/day)^a^	22.0 (20.7–22.9)	21.6 (20.0–22.7)	22.1 (21.0–23.0)	<0.01
Renovations or repairs^b^				0.69
No	3,189 (84.8)	1,493 (84.5)	1,696 (85.1)	
Yes	571 (15.2)	273 (15.5)	298 (14.9)	

KRW, Korean won; BMI, body mass index. Data are presented as ^a^median (25th percentile–75th percentile) or ^b^count (proportion, %).

Most participants were non-current smokers (83.0%) and did not engage in binge drinking (74.4%). The median BMI was 24.6 kg/m^2^ (IQR: 22.4–26.9). Participants spent a median of 22.0 h indoors daily (IQR: 20.7–22.9). Recent renovations or repairs were uncommon, with 84.8% reporting none. Men had significantly higher proportions of current smoking and binge drinking and a higher BMI than women (*p* < 0.01). Women spent more time indoors (*p* < 0.01).

### 3.2 Urinary creatinine-corrected phthalate metabolite concentrations

Urinary creatinine-corrected phthalate metabolite concentrations are shown in Table 3. Detection rates were 96.91% (MMP), 88.88% (MEP), 98.30% (MnBP), 97.85% (MBzP), 99.95% (MEHHP), 99.81% (MEOHP), 99.95% (MECPP), and 96.84% (MCPP). The GMs for urinary creatinine-corrected MMP, MEP, MnBP, MBzP, MEHHP, MEOHP, MECPP, and MCPP were 3.41, 4.63, 24.26, 1.10, 14.14, 7.69, 18.27, and 0.46 μg/g creatinine, respectively. Concentrations of all metabolites were higher in women than in men.

**TABLE 3:** Distribution of urinary creatinine-corrected phthalate metabolites (μg/g creatinine) in the study population.

Phthalate metabolites	Total (*n* = 3,760)			Male (*n* = 1,766)		Female (*n* = 1,994)		*P*-value
	Detection rate	GM (GSD)	Median (Q1–Q3)	GM (GSD)	Median (Q1–Q3)	GM (GSD)	Median (Q1–Q3)	
MMP	96.91%	3.41 (2.94)	3.96 (2.16–6.72)	2.89 (2.94)	3.33 (1.84–5.76)	3.94 (2.88)	4.62 (2.59–7.52)	<0.01
MEP	88.88%	4.63 (9.19)	3.77 (1.42–15.30)	3.93 (9.53)	3.10 (1.17–13.29)	5.35 (8.81)	4.49 (1.74–18.01)	<0.01
MnBP	98.30%	24.26 (3.03)	24.93 (15.59–42.28)	21.19 (3.20)	21.79 (14.02–35.71)	27.35 (2.83)	28.20 (17.48–45.88)	<0.01
MBzP	97.85%	1.10 (3.55)	1.06 (0.50–2.49)	1.00 (3.52)	1.00 (0.46–2.19)	1.19 (3.55)	1.14 (0.55–2.68)	<0.01
MEHHP	99.95%	14.14 (2.03)	13.98 (9.09–21.98)	12.65 (1.98)	12.58 (8.32–19.18)	15.61 (2.05)	15.55 (10.12–24.01)	<0.01
MEOHP	99.81%	7.69 (2.17)	7.78 (4.85–12.38)	6.64 (2.15)	6.78 (4.29–10.53)	8.75 (2.15)	8.88 (5.51–14.01)	<0.01
MECPP	99.95%	18.27 (1.99)	17.96 (11.75–27.96)	15.87 (1.93)	15.77 (10.60–23.91)	20.70 (1.99)	20.40 (13.41–31.89)	<0.01
MCPP	96.84%	0.46 (2.26)	0.45 (0.28–0.73)	0.38 (2.22)	0.38 (0.23–0.61)	0.54 (2.23)	0.51 (0.33–0.84)	<0.01

MMP, mono-methyl phthalate; MEP, mono-ethyl phthalate; MnBP, mono-*n*-butyl phthalate; MBzP, mono-benzyl phthalate; MEHHP, mono-(2-ethyl-5-hydroxyhexyl) phthalate; MEOHP, mono-(2-ethyl-5-oxohexyl) phthalate; MECPP, mono-(2-ethyl-5-carboxypentyl) phthalate; MCPP, mono-(3-carboxypropyl) phthalate; GM, geometric mean; GSD, geometric standard deviation; Q1, 25th percentile; Q3, 75th percentile.

Pearson’s correlation coefficients for the log-transformed, urinary creatinine-corrected metabolite concentrations are shown in Supplementary Figure 1. Significant positive correlations were observed among the metabolites. Strong correlations were found among MEHHP, MEOHP, and MECPP, which are metabolites of di(2-ethylhexyl) phthalate (DEHP) (*r* = 0.89–0.93). In contrast, MEP, a metabolite of diethyl phthalate, showed relatively weaker correlations with other metabolites (*r* = 0.11–0.22).

### 3.3 Associations of dietary and daily product-use factors with urinary phthalate metabolite patterns

The sRRR analysis revealed a single underlying pattern characterizing the associations between dietary and daily product-use factors and urinary phthalate metabolite levels. The model selectively retained key variables while effectively excluding 26 lifestyle-related factors that contributed minimally to the emergent structure.

As shown in Figure 1, the pattern was characterized by all eight metabolites exhibiting loadings in the same direction (MMP: +0.33, MEP: +0.10, MnBP: +0.27, MBzP: +0.34, MEHHP: +0.43, MEOHP: +0.44, MECPP: +0.46, and MCPP: +0.31). Several lifestyle factors exhibited loadings in the same direction as the outcome loadings, indicating that they were associated with higher urinary phthalate metabolite mixtures. For dietary consumption, mineral spring water (+0.08), grains (+0.08), and tap water (+0.06) showed substantial loadings in the same direction. For FCMs use, beverages in plastic pouches (+0.08) exhibited substantial loadings in the same direction.

**FIGURE 1:**
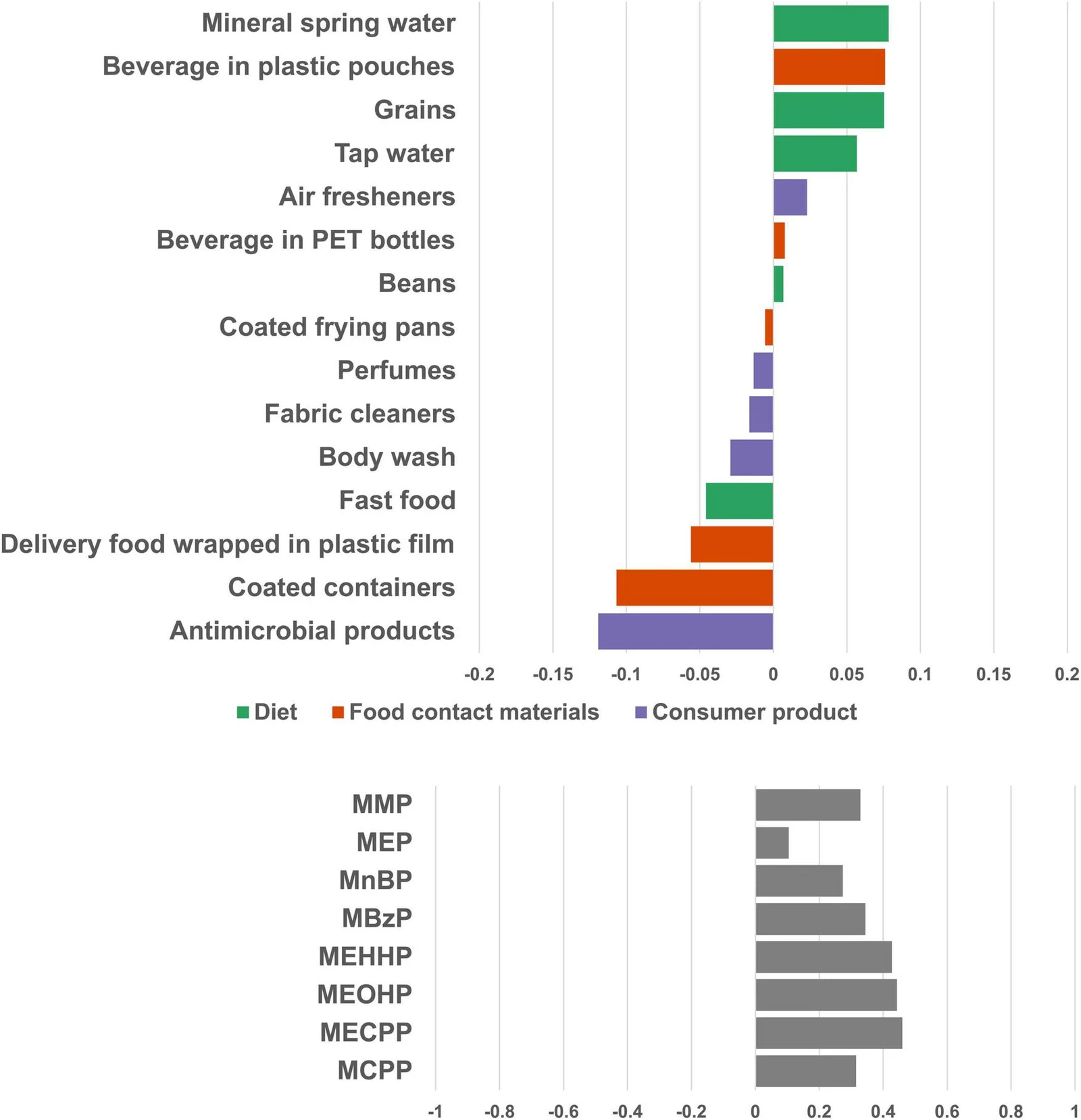
Contributions of dietary and daily product-use factors and urinary creatinine-corrected phthalate metabolites to the latent pattern. The *x*-axis represents the loading values derived from the sparse reduced-rank regression model. These values correspond to the predictor loadings (left singular vectors) and the response loadings (right singular vectors), where the magnitude indicates the strength of association with the latent pattern and the sign indicates the direction of association. The upper panel (predictor loadings) represents the association between exposure sources and the latent pattern, whereas the lower panel (response loadings) represents the association between urinary creatinine-corrected phthalate metabolites and the latent pattern. Signs were adjusted for interpretability. MMP, mono-methyl phthalate; MEP, mono-ethyl phthalate; MnBP, mono-*n*-butyl phthalate; MBzP, mono-benzyl phthalate; MEHHP, mono-(2-ethyl-5-hydroxyhexyl) phthalate; MEOHP, mono-(2-ethyl-5-oxohexyl) phthalate; MECPP, mono-(2-ethyl-5-carboxypentyl) phthalate; MCPP, mono-(3-carboxypropyl) phthalate.

Sex-stratified analyses revealed distinct latent patterns (Figure 2). Among men, dietary consumption, including grains, mineral spring water, and tap water, exhibited substantial loadings in the same direction as the eight metabolites. In contrast, among women, the use of FCMs and consumer products, such as beverages in plastic pouches and makeup products, exhibited substantial loadings in the same direction as the eight metabolites.

**FIGURE 2:**
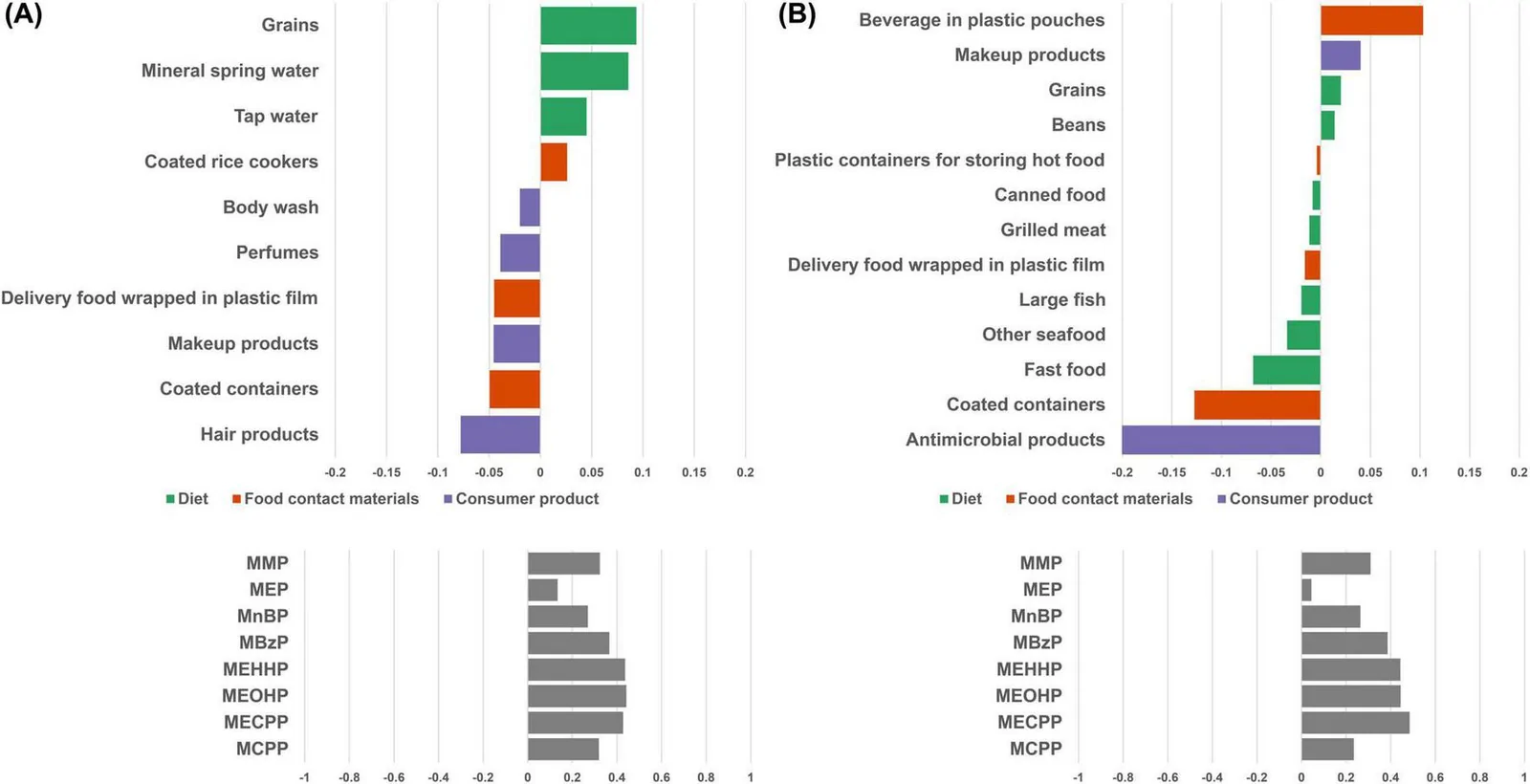
Contributions of dietary and daily product-use factors and urinary creatinine-corrected phthalate metabolites to the latent pattern in panel **(A)** males and **(B)** females. The *x*-axis represents the loading values derived from the sparse reduced-rank regression model. These values correspond to the predictor loadings (left singular vectors) and the response loadings (right singular vectors), where the magnitude indicates the strength of association with the latent pattern and the sign indicates the direction of association. The upper panels (predictor loadings) represent the association between exposure sources and the latent pattern, whereas the lower panels (response loadings) represent the association between urinary creatinine-corrected phthalate metabolites and the latent pattern. Signs were adjusted for interpretability. MMP, mono-methyl phthalate; MEP, mono-ethyl phthalate; MnBP, mono-*n*-butyl phthalate; MBzP, mono-benzyl phthalate; MEHHP, mono-(2-ethyl-5-hydroxyhexyl) phthalate; MEOHP, mono-(2-ethyl-5-oxohexyl) phthalate; MECPP, mono-(2-ethyl-5-carboxypentyl) phthalate; MCPP, mono-(3-carboxypropyl) phthalate.

## 4 Discussion

Using a nationally representative sample of Korean adults and an sRRR approach, this study identified a lifestyle-related pattern associated with higher urinary concentrations of multiple phthalate metabolites. Previous studies have reported phthalate exposure distributions and evaluated individual determinants (25–31); however, these were limited by their focus on isolated sources, separate evaluation of individual metabolites without adequately accounting for simultaneous co-exposure, and susceptibility to false positives from multiple testing (6). Given that phthalate exposure arises from multiple sources simultaneously (1–4, 16), such approaches cannot adequately characterize correlated lifestyle behaviors that drive co-exposure. In contrast, by jointly modeling multiple metabolites, our sRRR approach identified the lifestyle behaviors associated with the shared internal burden of multiple phthalates.

The central finding of this study was that, among routine behaviors, drinking mineral spring water and tap water, consuming grains, and using beverages packaged in plastic pouches were associated with elevated urinary phthalate metabolite levels. These findings align with the established understanding that ingestion is a predominant exposure route (5, 32). Notably, whereas conventional analyses often yield divergent source attributions across metabolites, complicating prioritization, our approach identified a common subset of potential sources that contribute to multiple metabolites. This integrated source profile offers population-specific empirical evidence relevant to public health recommendations and regulatory consideration (4, 5).

Drinking mineral spring and tap water was associated with higher levels of urinary phthalate metabolite mixtures. Contamination can occur at multiple stages within the water supply chain (33, 34). For instance, raw water may be affected by inadequately treated wastewater, poor waste management, or wastewater utilization for agricultural irrigation (34, 35). Furthermore, the supply system can exacerbate contamination (33, 34). Conventional drinking water treatment plants, primarily designed to remove suspended solids, bacteria, and natural organic matter, are ineffective against phthalates (34, 36), and residues often persist (36). Secondary contamination can occur downstream through leaching from plastic pipelines and point-of-use treatment units (34). Notably, in Korea, only DEHP is regulated in drinking water, with a permissible limit of 80 μg/L, higher than the standards set by the U.S. Environmental Protection Agency and the World Health Organization, and DEHP removal rates in Korean water treatment facilities are reported to be ineffective, ranging from 8.2% to 33.4% (36).

Grain consumption, a major component of the Korean diet (37), was also associated with higher levels of urinary phthalate metabolite mixtures. Although we did not measure phthalate concentrations in participants who consumed specific grains, the observed association may be partly explained by contamination arising from agricultural practices, including plastic mulch films that can leach chemicals into the soil and subsequently into crops (38–40). Another possible explanation is environmental deposition from air and dust during processing and migration from packaging materials, such as plastic bags or containers used in storage and transport (41, 42).

The consumption of beverages packaged in plastic pouches was also associated with higher levels of urinary phthalate metabolite mixtures, which may be related to the widespread use of phthalates as plasticizers in food-contact materials and their potential migration into foods and beverages during processing and storage (41, 42). Plastic pouches, which often undergo heat sealing and prolonged storage (43, 44), may have a greater potential for leaching, given that migration rates from FCMs are influenced by temperature, contact duration, and the physicochemical properties of the food matrix (41, 45). Under Korea’s common manufacturing standard for FCMs, DEHP should not be used if migration into food may occur (46). For articles intended for infants and young children, as well as polyvinyl chloride (PVC) materials, regulations also apply to several other phthalate species (46).

Furthermore, the lifestyle factors most prominently associated with urinary phthalate metabolite mixtures differed by sex: among men, these were the consumption of mineral spring water, tap water, and grains, whereas among women, these were the consumption of beverages packaged in plastic pouches and the use of makeup products. The latter likely reflects sex-specific patterns of makeup product use, with women typically using more makeup products than men. These sex-specific differences underscore the need for tailored approaches to exposure reduction that account for differential product-use patterns.

This study has several strengths. First, the application of sRRR enabled simultaneous consideration of diverse lifestyle factors while mitigating multicollinearity across predictors (17, 22), thereby characterizing phthalate exposure as the result of key combined behaviors. Furthermore, by jointly modeling multiple metabolites, this approach offered a more integrated representation of real-world exposure and enabled the identification of a lifestyle pattern contributing to the overall phthalate burden, which provides more interpretable insights for decision-making. Second, the use of a large, nationally representative sample ensured that the findings were robust and highly generalizable to Korean adults. Third, the detailed and well-structured questionnaire of KoNEHS enabled comprehensive assessments of multiple daily lifestyle factors, including diet, FCMs, and consumer products, allowing for a more complete understanding of phthalate exposure pathways.

However, this study has several limitations. First, the cross-sectional design limited our ability to draw causal inferences. Second, exposure assessments based on questionnaire data and urinary biomarkers have inherent limitations. Dietary consumption and daily product use were self-reported and may therefore be subject to recall bias and measurement error. Moreover, questionnaire-based dietary and lifestyle variables used in this study represent habitual long-term behaviors, whereas urinary metabolites capture recent exposure owing to the short biological half-lives of phthalates. This temporal mismatch may lead to exposure misclassification and complicate causal interpretation; accordingly, the findings should be understood as identifying habitual exposure patterns rather than direct short-term dose determinants. Third, potential seasonal variation in phthalate exposure could not be accounted for, as information on the month or season of biospecimen collection was unavailable to adjust for such confounding. Fourth, phthalate concentrations were not directly measured in the drinking water, foods, or products reported by participants. As phthalate concentrations in potential sources may vary substantially, the factors identified as being associated with urinary phthalate mixture exposure represent behaviors or lifestyle characteristics associated with exposure rather than confirmed direct exposure sources. Moreover, without source- or product-specific chemical measurements, the precise origins and pathways of contamination remain undetermined. For instance, it is unclear whether drinking water contamination stems from raw source water, the water supply system, or packaging materials. Future studies should incorporate longitudinal designs, integrate environmental and product-specific measurements with biomonitoring, and identify the origins of contamination to better inform regulatory actions.

The eight metabolites measured in this study characterize exposure to the six principal parent phthalates, although MCPP is not specific to a single parent compound and is commonly attributed to di-*n*-octyl phthalate (DnOP) (6, 47). MMP, MEP, MnBP, and MBzP are commonly used as biomarkers of dimethyl phthalate (DMP), diethyl phthalate (DEP), di-*n*-butyl phthalate (DnBP), and benzyl butyl phthalate (BBzP), respectively, whereas MEHHP, MEOHP, and MECPP are oxidative metabolites of DEHP (6, 14). These parent phthalates have distinct uses and exposure profiles. DMP and DEP have been used as solvents or fragrance fixatives in PCPs and other non-PVC applications, whereas DnBP has been used in PCPs, particularly in nail polish, as well as in lacquers, varnishes, adhesives, printing inks, and coatings (41, 48, 49). DEHP has been widely used as a plasticizer in PVC-containing consumer products, including certain FCMs, whereas BBzP spans both domains, being used in paints, adhesives, and sealants and as a plasticizer in PVC articles ranging from vinyl flooring to some FCMs (41, 48, 49). Consistent with these use patterns, consumer products and indoor sources are important contributors to DMP and DEP exposure, with DEP exposure being particularly associated with fragranced PCPs (48, 49), whereas food contributes substantially to DnBP and DEHP exposure, reflecting diffuse environmental contamination together with migration into food from packaging adhesives and printing inks and from processing equipment (41, 48, 49). For BBzP, consumer products and indoor sources as well as food have been identified as important contributors (48, 50). MCPP is a nonspecific oxidative metabolite. In experimental rats, it was formed most abundantly after DnOP exposure but was also detected after exposure to di-isooctyl phthalate, di-isononyl phthalate (DiNP), di-isodecyl phthalate (DiDP), DEHP, and DnBP, and its specificity as a human biomarker remains uncertain (47). DiNP and DiDP have increasingly been used as substitutes for DEHP in plasticized products (48, 50, 51).

Korean regulations cover these compounds unevenly (46). Among the phthalates analyzed in this study, DEHP is the only one with an established national drinking water monitoring criterion (36). Under common manufacturing standards for FCMs, DEHP is prohibited when migration into food may occur (46). DnBP and BBzP are additionally prohibited in the manufacture of utensils, containers, and packages intended for infants and young children, and PVC-specific migration limits apply to DnBP, BBzP, DEHP, DnOP, and the sum of DiNP, and DiDP (46). In cosmetics, the combined concentrations of DnBP, BBzP, and DEHP are limited to 100 μg/g (46). DMP and DEP are not listed in drinking water monitoring, FCM, or cosmetics provisions (36, 46). Regulatory attention is thus concentrated on DEHP, whereas the lifestyle pattern identified here, although led by the DEHP metabolites, was also appreciably associated with the remaining metabolites. This indicates that the internal burden associated with these behaviors extends well beyond the phthalates that current provisions primarily target and suggests that the expansion of the list of regulated phthalate species should be considered (52).

In summary, this study explored the dietary and daily product-use factors associated with exposure to multiple phthalates among Korean adults. Using a predictive and exploratory analytical framework, we provide a population-level characterization of the patterns of dietary consumption and daily product use associated with urinary phthalate metabolite levels. In this nationally representative sample, drinking water, grains, and beverages in plastic pouches made the largest contributions to the lifestyle-related pattern associated with higher urinary phthalate metabolite concentrations. These findings provide empirical grounds for further investigation through prospective studies that integrate biomonitoring with environmental and product-specific measurements to clarify source-specific exposure pathways and inform evidence-based exposure-reduction strategies. If future studies confirm causal relationships between the lifestyle factors identified in our study and phthalate exposure, those findings could help inform policies for reducing cumulative exposure, including regulations for drinking water and FCMs, improvements in water treatment infrastructure, and expansion of the list of regulated phthalate species (52). Even though definitive recommendations await further confirmation, consuming filtered or bottled water instead of tap or mineral spring water and reducing reliance on plastic-based FCMs to minimize leaching may be considered as precautionary approaches to help lower exposure (52–54).

## Data Availability

The datasets analyzed in this study are available from the Korean National Environmental Health Survey (KoNEHS) and can be obtained from the National Institute of Environmental Research (NIER) upon reasonable request. Additional information is available at https://www.ehtis.or.kr/. Requests to access these datasets should be directed to knehs@korea.kr.
