# Supplementary material for: Identifying dietary and daily product-use patterns explaining joint variation in multiple urinary phthalate metabolites via sparse reduced-rank regression
Source: Front Nutr. 2026 Sep 22;13:1929257. doi: 10.3389/fnut.2026.1929257 (PMC13638432; doi:10.3389/fnut.2026.1929257)
Supplement: Supplementary file 1 [file Data_Sheet_1.docx]

Supplementary Material

# 1 Supplementary Data

This file contains the supplementary figure referenced in the main manuscript. Supplementary Figure 1 presents the pairwise correlations among the eight urinary creatinine-corrected phthalate metabolites analyzed in the study.

# 2 Supplementary Figures and Tables

## 2.1 Supplementary Figures

**Supplementary Figure 1.** Correlations among concentrations of urinary creatinine-corrected phthalate metabolites. MMP, mono-methyl phthalate; MEP, mono-ethyl phthalate; MnBP, mono-n-butyl phthalate; MBzP, mono-benzyl phthalate; MEHHP, mono-(2-ethyl-5-hydroxyhexyl) phthalate; MEOHP, mono-(2-ethyl-5-oxohexyl) phthalate; MECPP, mono-(2-ethyl-5-carboxypentyl) phthalate; MCPP, mono-(3-carboxypropyl) phthalate.
